# Human α-defensin (DEFA) gene expression helps to characterise benign and malignant salivary gland tumours

**DOI:** 10.1186/1471-2407-12-465

**Published:** 2012-10-11

**Authors:** Jochen Winter, Annette Pantelis, Dominik Kraus, Jan Reckenbeil, Rudolf Reich, Soeren Jepsen, Hans-Peter Fischer, Jean-Pierre Allam, Natalija Novak, Matthias Wenghoefer

**Affiliations:** 1 Department of Periodontology, Operative and Preventive Dentistry, University of Bonn, Welschnonnenstr, 1753111, Bonn, Germany; 2 Waldkrankenhaus, Waldstrasse 73, 53177, Bonn, Germany; 3 Department of Prosthodontics, Preclinical Education, and Material Science, University of Bonn, Welschnonnenstr, 1753111, Bonn, Germany; 4 Department of Oral & Maxillofacial Plastic Surgery, University of Bonn, Sigmund-Freud-Str. 25, 53105, Bonn, Germany; 5 Department of Pathology, University of Bonn, Bonn, Germany; 6 Department of Dermatology and Allergy, University of Bonn, Sigmund-Freud-Str. 25, 53105, Bonn, Germany

**Keywords:** DEFA 1/3, DEFA 4, Salivary gland, Tumour, Defensins

## Abstract

**Background:**

Because of the infrequence of salivary gland tumours and their complex histopathological diagnosis it is still difficult to exactly predict their clinical course by means of recurrence, malignant progression and metastasis. In order to define new proliferation associated genes, purpose of this study was to investigate the expression of human α-defensins (DEFA) 1/3 and 4 in different tumour entities of the salivary glands with respect to malignancy.

**Methods:**

Tissue of salivary glands (n=10), pleomorphic adenomas (n=10), cystadenolymphomas (n=10), adenocarcinomas (n=10), adenoidcystic carcinomas (n=10), and mucoepidermoid carcinomas (n=10) was obtained during routine surgical procedures. RNA was extracted according to standard protocols. Transcript levels of DEFA 1/3 and 4 were analyzed by quantitative realtime PCR and compared with healthy salivary gland tissue. Additionally, the proteins encoded by DEFA 1/3 and DEFA 4 were visualized in paraffin-embedded tissue sections by immunohistochemical staining.

**Results:**

Human α-defensins are traceable in healthy as well as in pathological altered salivary gland tissue. In comparison with healthy tissue, the gene expression of DEFA 1/3 and 4 was significantly (p<0.05) increased in all tumours – except for a significant decrease of DEFA 4 gene expression in pleomorphic adenomas and a similar transcript level for DEFA 1/3 compared to healthy salivary glands.

**Conclusions:**

A decreased gene expression of DEFA 1/3 and 4 might protect pleomorphic adenomas from malignant transformation into adenocarcinomas. A similar expression pattern of DEFA-1/3 and -4 in cystadenolymphomas and inflamed salivary glands underlines a potential importance of immunological reactions during the formation of Warthin’s tumour.

## Background

Salivary gland tumours are a rare tumour entity: they have a prevalence of 2-3% among the head and neck cancers and a proportion of less than 0.5 percent of all malignant tumours. Their majority is benign, but malignant salivary gland tumours occur in 15 to 32 percent of the patients. In the most cases they derive from the six major salivary glands (parotid, submandibular, and sublingual gland), most commonly from the parotid gland in up to 80 percent of all cases [1].

The exact histopathological diagnosis is complicated by a wide variety of different morphologic features. Salivary gland tumours might be subdivided by their main histopathological features into tumours with a) myoepithelial components, b) basaloid components, c) epithelial components, d) lymphatic components and e) pleomorphic adenomas [2].

As salivary gland tumours are not very frequent and their histopathological diagnosis is complex, at present time it is difficult to exactly predict their clinical course by means of recurrence, malignant progression and metastasis. Therefore molecular biology has moved into the focus of tumour research. Like in other epithelial tumours a number of proliferation associated antigens as Ki-67, proto-oncogenes as bcl-2, tumour suppressor genes as p53 or p21 and the overexpression of growth-factor binding receptors as HER-2 have been identified as important factors in the malignant progression of these tumors [3], but disappointingly only a few candidate genes and their transcripts have been clearly associated with the malignant transformation and progression of benign salivary gland tissue into a malignant tumour. As pleomorphic adenomas have the tendency in up to 4% of the cases to transform into an adenocarcinoma, they are ideal subjects to investigate and characterise altered genes in the molecular sequence adenoma to carcinoma. Interesting new candidate genes which might help to a better understanding of salivary gland tumours are human defensins [4-6].

### Defensins

Antimicrobial peptides (AMPs) were first described in the coherency of host defence, but feature besides their antimicrobial activity a wide variety of functions in numerous cellular processes. One subfamily of mammalian AMPs are defensins, which are important components of the innate immune system. Defensins are positively charged peptides with molecular weights ranging from 3.5 to 6.5 kDa. They share a framework of six disulphide-linked cysteine (cys) residues, thus forming a characteristic β-sheet structure under physiological conditions. A number of positively charged amino acids cause their typical cationic features and is, in combination with their physical structure, responsible for their ability to disintegrate membranes of gram-positive and gram-negative bacteria as well as yeasts and certain viruses. Defensins have been detected in different tissues as the epithelia of the oral cavity, the gastrointestinal and respiratory tract, the urinary tract and the vagina as well as in the salivary glands [7-11].

By their physical structure human defensins could be divided into two subgroups: α- and β-defensins, which both consist of a triple-stranded β-sheet structure, but mainly differ in the pairing of their cystine-disulphide bridges. Cysteine residues in α-defensins are linked between 1-6/2-4/3-5, whereas β-defensins share a 1-5/2-4/3-6 cys-cys pattern. By this characteristic pairing of disulphide bridges, four α-defensins (DEFA) were described, which have been isolated from the granules of polymorphonuclear neutrophil leukocytes. Because of their distribution in the granules of the leucocytes, they were named human neutrophil peptides 1-4 (HNP1-4). Additionally two α-defensins, DEFA 5 and 6 were isolated from intestinal Paneth cells. The genes encoding for the DEFAs are located on chromosome 8, remarkably DEFA 1 differs from DEFA 3 only by one amino acid [7,8,10-13].

In recent studies, human beta-defensins (hBDs) were investigated in head and neck cancers - and there seems to be a correlation between chronic inflammation and cancers of the oral cavity, like tongue carcinomas [14,15].

This observation is consistent with prior studies concerning the involvement of hBD-1 in the carcinogenesis of epithelial tumours of the urogenital tract: Refering to healthy epithelium showing an intact hBD-1 gene expression, there was a cancer-specific loss of hBD-1 gene expression in 90% of renal clear cell carcinomas and 82% of malignant prostate cancers [16,17]. Recent studies transferred this observation towards oral squamous cell carcinomas (OSCC) in which the hBD-1 gene expression is significantly (50-fold) reduced in comparison with healthy gingiva [18]. As human defensins are involved in chronic periodontal inflammation as well as carcinogenesis of oral tumours, it is an interesting new hypothesis that they might act as key-molecules in the inflammation-tumour-sequence [4-6,17,19-22]. The gene *DEFB1* encoding for hBD-1 has been identified as a major periodontitis-associated gene [23] with functions in local host defence but also seems to be an important factor in proliferation control of oral cancers [4-6,17,19-21]. *In vitro* hBD-1 inhibits the proliferation of oral squamous cell carcinoma cells [16-19], whereas hBD-2 and -3 promote their proliferation [19,20]. This observation could also be made in osteosarcoma cell lines, where hBD-2 and -3 enhance proliferation [24]. Furthermore hBD-1, -2 and -3 cross-regulate their own gene expression in OSCCs *in vitro*[19,20].

So we gained some insight recently in the role which human β-defensins might play in the carcinogenesis of head and neck cancers – maybe they are the link between chronic inflammation and epithelial tumour initiation. Because of the structural and functional similarities between α- and β-defensins, α-defensins could have similar properties in these tumours as β-defensins.

## Methods

### Tissue sampling

In this study, tissue of inflamed salivary glands (n=10), pleomorphic adenomas (n=10), cystadenolymphomas (n=10), adenocarcinomas (n=10), adenoidcystic carcinomas (n=10), and mucoepidermoid carcinomas (n=10) was investigated and compared with healthy salivary gland tissue as reference. The average age at diagnosis was 53.8 years. The healthy salivary gland tissue was collected from patients with head and neck tumours during the surgical procedure of neck dissection; those patients were neither irradiated nor received chemotherapy. Procedures involving the human tissue sampling collection followed a protocol approved by the ethical board of the University of Bonn. All patients had been informed about the study and had signed a letter of informed consent.

### Immunohistochemistry

After formalin fixation, the tissue samples were embedded in paraffin and cut with a standard microtome (Reichert-Jung, Heidelberg, Germany) into 2μm sections. Tissues for light microscopic evaluation were stained with Hematoxylin and Eosin (HE) and the diagnosis was confirmed by a pathologist.

Formalinfixed, paraffin-embedded tissue sections were used for immunohistochemical staining. After deparaffinization and rehydration, the slides were washed in Tris-buffered saline (TBS) containing 1% bovine serum albumin (BSA). Endogenous peroxidase activity was quenched by incubating the slides in a methanolic solution of 0.3% hydrogen peroxide. Tissue samples were blocked for unspecific binding with 1% BSA in TBS for 1 hour at room temperature. Incubation with primary antibodies rabbit anti-DEFA 1/3 and -DEFA 4 (Santa Cruz, Heidelberg, Germany) diluted 1:200 in 1% TBS-buffered BSA was performed overnight at 4°C.

The slides were then washed in TBS buffer, the HRP-conjugated secondary antibody, goat anti-rabbit (Dianova, Hamburg, Germany), was added and the slides were incubated at room temperature for 45 minutes. Afterwards, the slides were washed in TBS buffer and incubated with diaminobenzidine (DAB) as substrate and counterstained with hematoxylin (Merck Eurolab, Dietikon, Switzerland). Negative controls without primary antibody were included in each experiment to verify antibody specificity. The immuno-staining for DEFA 1/3 and DEFA 4 was analyzed using a Zeiss Axio-Imager A.1 microscope (Zeiss, Oberkochen, Germany).

### RNA extraction and first-strand cDNA synthesis

RNA was prepared only from isolated tumour sections using the “RNeasy Protect Mini Kit” (Qiagen, Hilden, Germany). Circumjacent non-tumourigenic tissues were cut-off. The preparation of tumour sections was microscopically verified. First-strand cDNA synthesis was performed using "iScript™ Select cDNA Synthesis Kit" (Bio-Rad, Munich, Germany) with oligo(dT) primers according to the manufacturer’s protocol.

### Quantitative realtime-PCR

Differential gene expression was analyzed by realtime-PCR with the iCycler® Thermal Cycler (Bio-Rad, Munich, Germany). SYBR® Green served as fluorophor for online-monitoring of generated PCR-products. All primers were synthesized and specified by Metabion (Metabion, Martinsried, Germany. Primer sequences are presented in Table 1. Realtime-PCR was performed as previously described [4,6,19,21,22,24]. An appropriate amount of cDNA was added to a mastermix containing primers and iQ™ SYBR® Green Supermix (Bio-Rad, Munich, Germany). Cloned PCR-products derived from the specific primers served as positive controls for the PCR, while water was used as negative control. Every set of experiment was carried out with cDNA of the same sample to exactly compare the expression of the different genes of interest. Glyceraldehydephosphate-dehydrogenase (GAPDH) served as reference to normalize the crossing point (CP).

**Table 1:** Primer sequences with corresponding annealing temperatures, efficiencies, and product length in basepairs (bp) used for realtime PCR

**Gene**	**Primer sequences (sense/antisense)**	**Efficiency**	**Annealing temperature (°C)**	**Product length (bp)**
**GAPDH**	5′;-TGGTATCGTGGAAGGACTCA-3′;	1.93	67	132
	5′;-CCAGTAGAGGCAGGGATGAT-3′;			
**DEFA1/3**	5′;-ATGAGGACCCTCGCCATCCTTGCT-3′;	2.17	69	285
	5′;-TCAGCAGCAGAATGCCCAGCGTCTTCCC-3′;			
**DEFA4**	5′;-GTCTGCTCTTGCAGATTAGTATTCTG-3′;	1.98	69	105
	5′;-TTAATCGACACGCGTGCAGCAGTAT-3′;			

PCR efficiencies for every set of primers were determined with dilution series of primer specific cloned PCR-products at the corresponding annealing temperature. Efficiency stands for the performance to amplify a cDNA template. Efficiency values of 2.0 means an amplification of 100%.

Relative differential gene expression was calculated using the method described by Pfaffl [25]. PCR-efficiencies were determined with dilution series. Efficiency (E) is defined with E = 10^-1/slope^[25]. Primer efficiencies and corresponding annealing temperatures are depicted in Table 1.

### Statistical analysis

In case of multiple comparisons of the investigated groups one-way ANOVA with Dunnett′;s post-test was performed. All statistical analyses were performed by GraphPad Prism version 5.00 for Windows, GraphPad Software (San Diego, USA). Significant differences (p<0.05) compared to control are marked with asterisks (*).

## Results

### Human α-defensins are traceable in healthy as well as pathological altered salivary gland tissue

In comparison with healthy tissue (set 1 as baseline), the gene expression of DEFA-1/3 and -4 was significantly (p<0.05) increased in all tumours. The increase for DEFA 1/3 was in pleomorphic adenomas: 2.64-fold, in cystadenolymphomas: 146.9-fold, in adenocarcinomas as well as in adenoidcystic carcinomas: 48.47-fold and in mucoepidermoid carcinomas: 27.84-fold.

The increase of DEFA 4 was in cystadenolymphomas: 48.2-fold, in adenocarcinomas: 13.9-fold, adenoidcystic carcinomas: 14.5-fold and in mucoepidermoid carcinomas: 4.6-fold.

The increase of DEFA 1/3 and DEFA 4 in inflamed salivary gland tissue (146.9 and 30.3-fold respectively) was comparable with cystadenolymphomas (146.9 and 48.2-fold respectively).

Only in pleomorphic adenomas a 3.4-fold decrease in DEFA 4 gene expression was evident.

**Figure 1:**
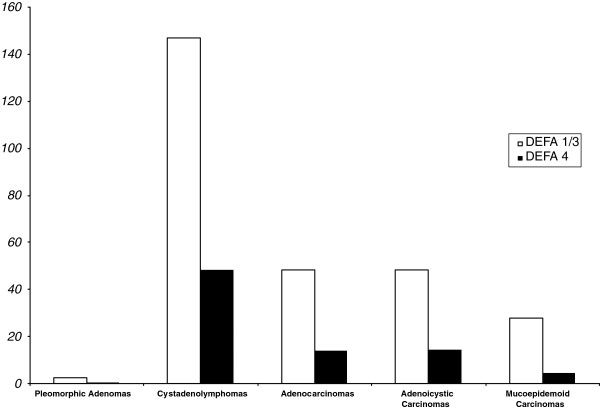
Differential gene expression of DEFA 1/3 and DEFA 4 in different salivary gland tumors in comparison with healthy salivary gland tissue as baseline (= 1).

The results for the relative gene expressions of DEFA 1/3 and DEFA 4 are depicted in Table 2, the results for the comparison of all entities with healthy salivary gland tissue are shown in Table 3 and visualized in Figure 1.

**Table 2:** Relative gene expression (mean values with standard deviations) of DEFA 1/3 and DEFA 4 compared to GAPDH in healthy salivary glands, pleomorphic adenomas, cystadenolymphomas, adenocarcinomas, adenoidcystic carcinomas, and mucoepidermoid carcinomas (n=10 each)

**Entity**	**DEFA 1/3**	**DEFA 4**
**Healthy salivary glands**	0.0237 (0.0025)	0.00056 (0.0001)
**Pleomorphic adenomas**	0.0625 (0.023)	0.00016 (0.00003)
**Cystadenolymphomas**	3.4822 (1.69)	0.027 (0.0057)
**Adenocarcinomas**	1.1487 (0.62)	0.0078 (0.001)
**Adenoidcystic carcinomas**	1.1487 (0.51)	0.0081 (0.001)
**Mucoepidermoid carcinomas**	0.6597 (0.32)	0.00258 (0.0003)

**Table 3:** Differential gene expression analysis of DEFA 1/3 and DEFA 4 in pleomorphic adenomas, cystadenolymphomas, adenocarcinomas, adenoidcystic carcinomas, and mucoepidermoid carcinomas compared to healthy salivary gland tissue (set as baseline = 1) – significant differences (p<0.05) are marked with asterisks (*)

**Entity**	**DEFA 1/3**	**DEFA 4**
**Pleomorphic adenomas**	2.64*	0.29*
**Cystadenolymphomas**	146.9*	48.2*
**Adenocarcinomas**	48.47*	13.9*
**Adenoidcystic carcinomas**	48.47*	14.5*
**Mucoepidermoid carcinomas**	27.84*	4.6*

**Figure 2:**
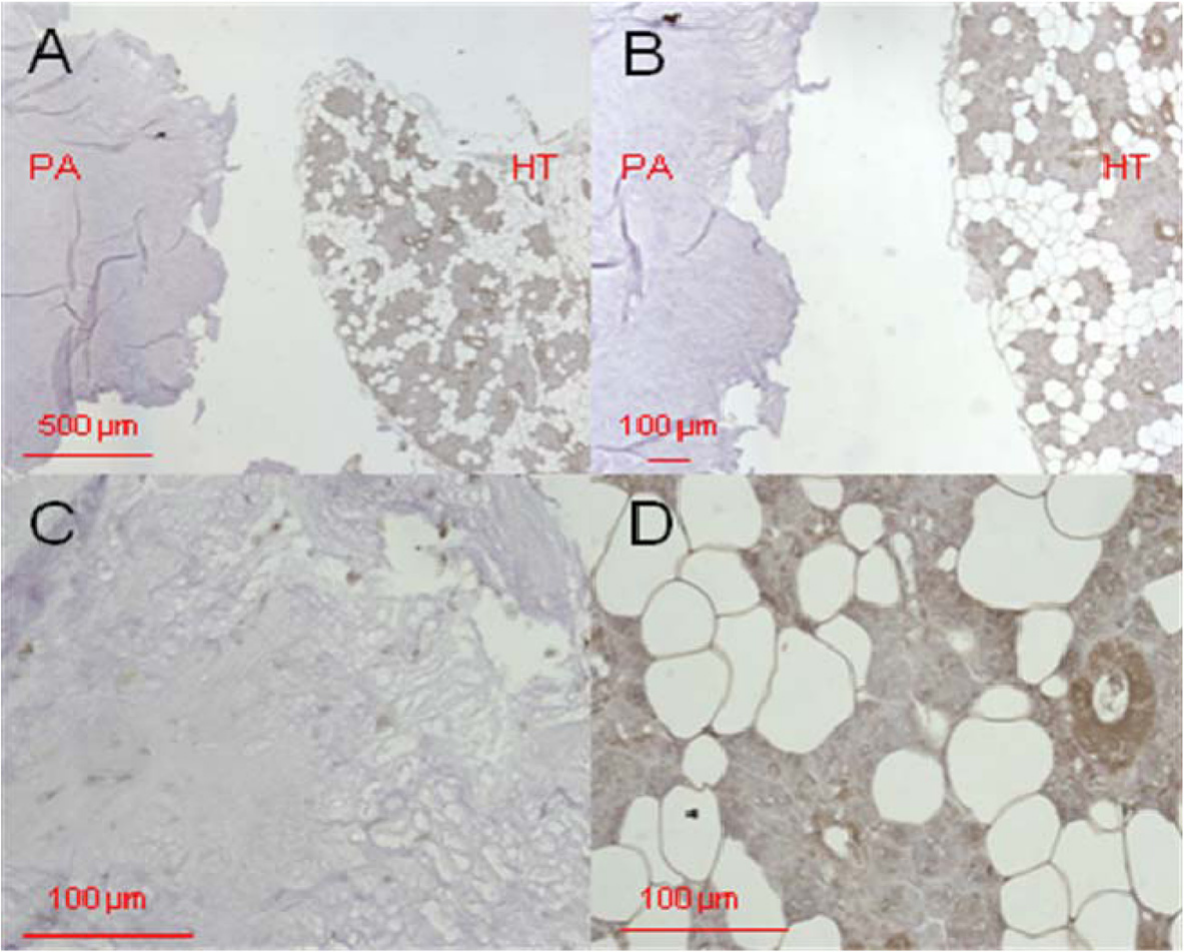
**DEFA1/3 immunostaining in pleomorphic adenoma tissue.** Primary magnification 5-fold (**A**), 10-fold (**B**), and 40-fold (**C**, **D**). The tissue section on the left in A and B (designated with "PA") shows the pleomorphic adenoma status, the section on the right of A and B (designated as "HT") shows healthy tissue isolated from the biopsy. C represents the pleomorphic adenoma, D the healthy section.

**Figure 3:**
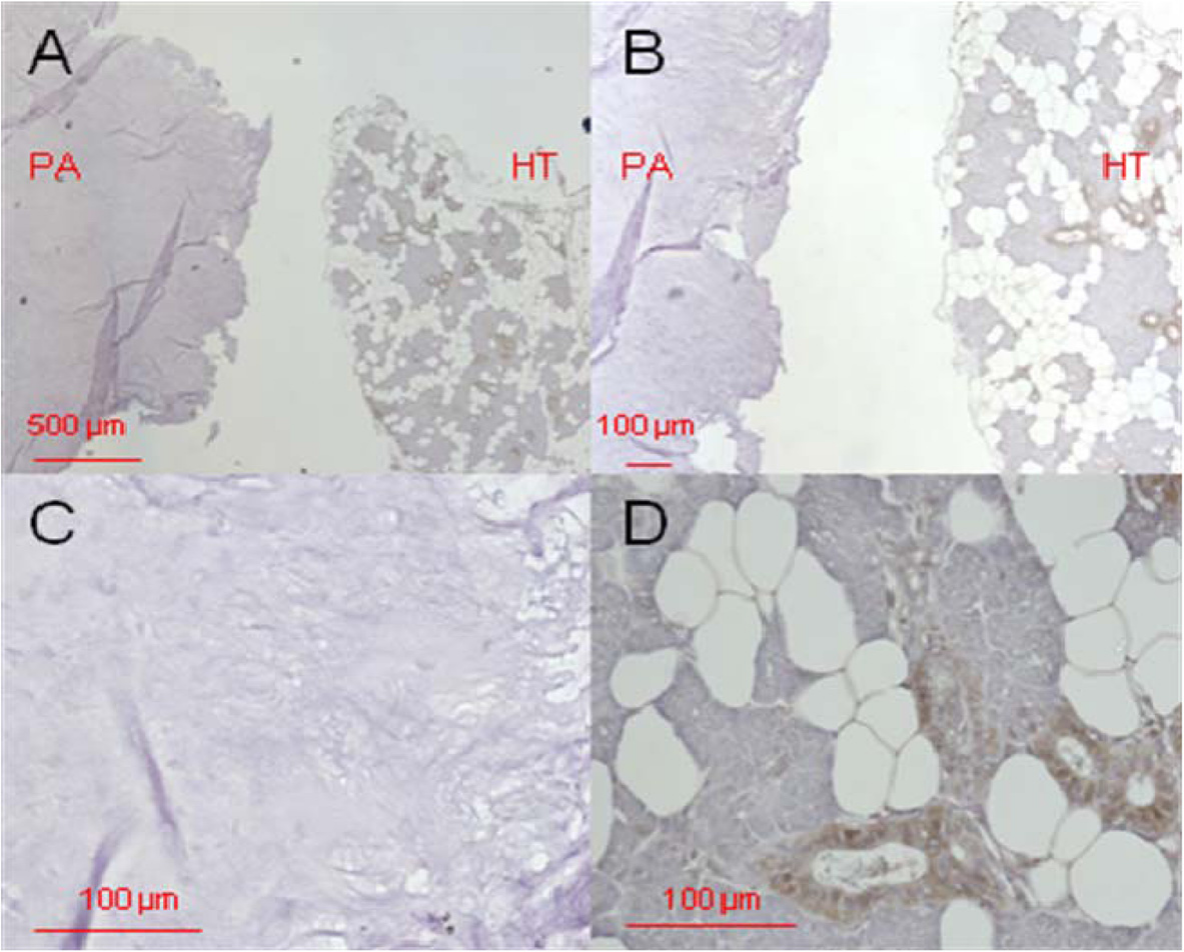
**DEFA4 immunostaining in pleomorphic adenoma tissue.** Primary magnification 5-fold (**A**), 10-fold (**B**), and 40-fold (**C**, **D**). The tissue section on the left in A and B (designated with "PA") shows the pleomorphic adenoma status, the section on the right of A and B (designated as "HT") shows healthy tissue isolated from the biopsy. C represents the pleomorphic adenoma, D the healthy section.

Figure 2 shows the immunostaining for DEFA 1/3, Figure 3 the immunostaining for DEFA 4 in pleomorphic adenoma tissue.

**Figure 4:**
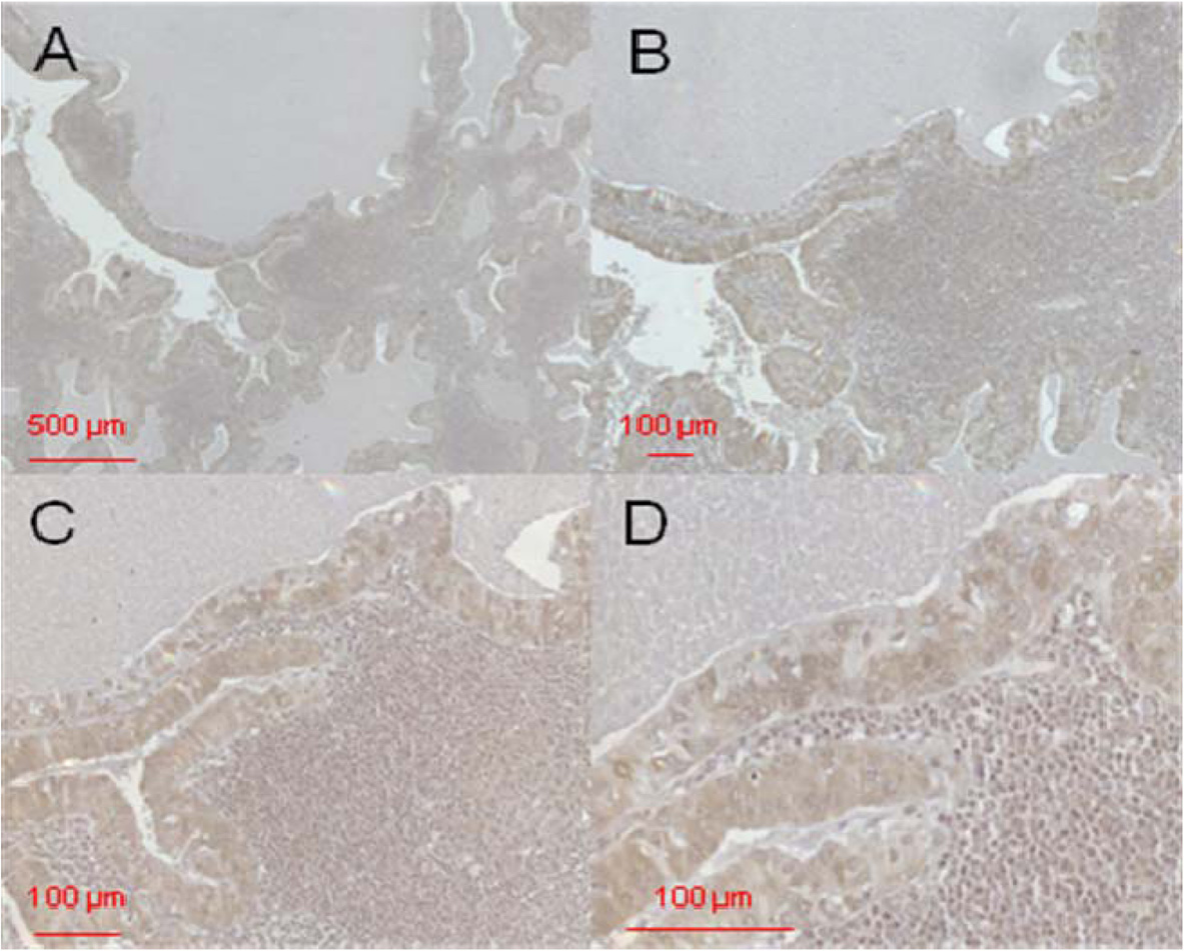
**Immunostaining of DEFA1/3 in cystadenolymphoma.** Primary magnification 5-fold (**A**), 10-fold (**B**), 20-fold (**C**), and 40-fold (**D**).

**Figure 5:**
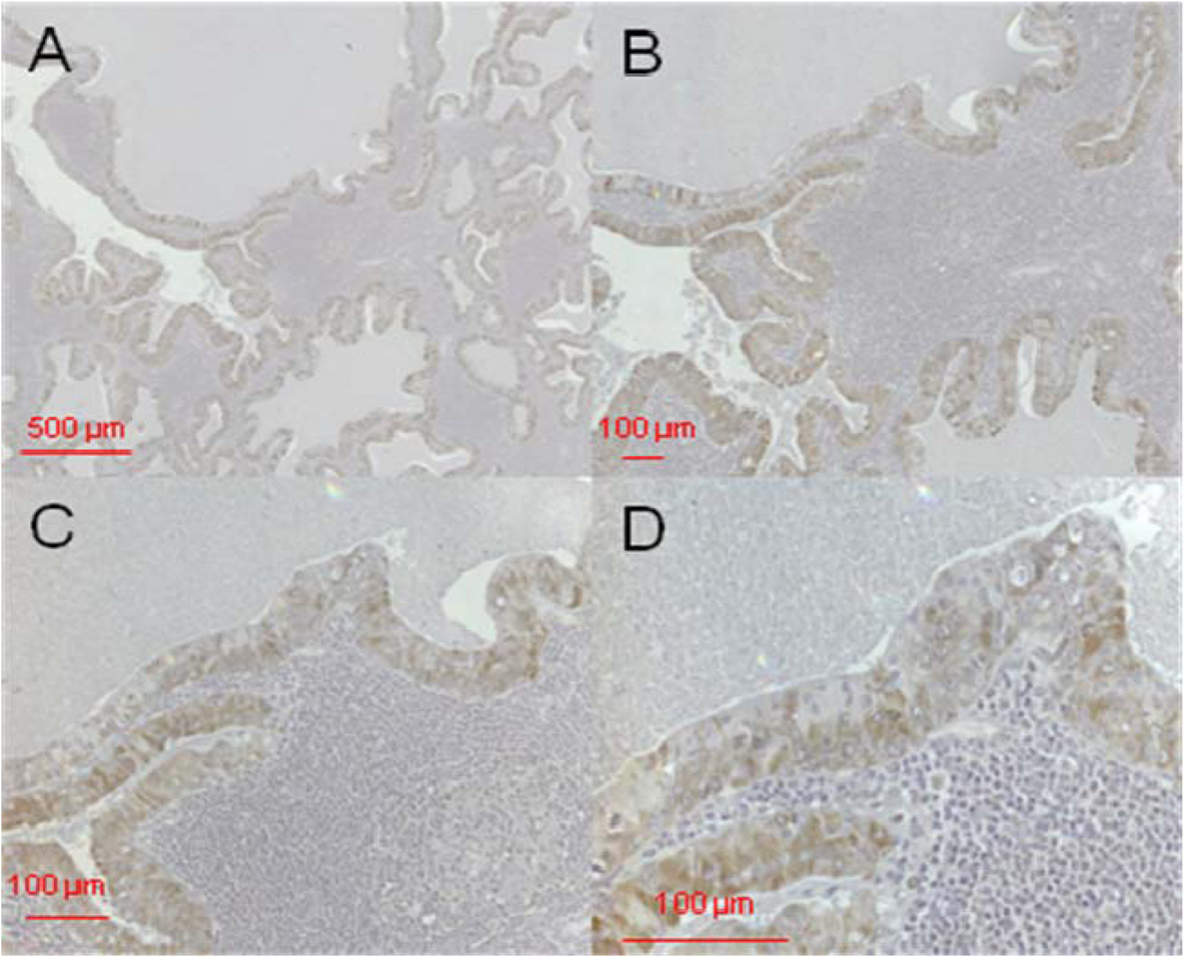
**Immunostaining of DEFA4 in cystadenolymphoma.** Primary magnification 5-fold (**A**), 10-fold (**B**), 20-fold (**C**) and 40-fold (**D**).

Figure 4 shows the immunostaining for DEFA 1/3, Figure 5 the immunostaining for DEFA 4 in cystadenolymphoma tissue. Primary magnification of all images was: A=5-fold, B=10-fold, C=20-fold and D=40-fold.

## Discussion

In the present study the gene expression of DEFA 1/3 ad DEFA 4 was analyzed in healthy salivary gland tissue in comparison with different benign and malignant salivary gland tumours and inflamed salivary gland tissue. Additionally the peptides were visualized by immunostaining.

Both α-defensins were detectable in healthy tissue, inflamed tissue as well as tumour tissue. In comparison with healthy salivary gland tissue, which was taken as baseline, the gene expression of DEFA 1/3 and DEFA 4 was altered in all investigated tissues – but most interesting were the findings in cystadenolymphomas and pleomorphic adenomas (Tables 2 and 3):

Cystadenolymphomas, although first mentioned by Hildebrad in 1885 [26] and more precisely described by the German pathologist Albrecht in 1910 [27] are named “Warthin tumor” after the American pathologist Aldred Warthin who published 34 years later the first two cases of this entity in American literature [28]. Cystadenolymphomas are composed of a bilayered oncocytic and basaloid epithelium, forming cystic structures, papillae and glands that are accompanied by a dense lymphoid stroma [29-31]. Their origin is still a kind of cryptic today: some authors think that they might develop of heterotopic epithelial tissue enclosed in the lymph nodes within the parotid gland whereas others believe that they are adenomas with a lymphocytic infiltration [29-31]. But Interestingly recent studies have shown, that the above mentioned epithelial component is polyclonal, which means that it does not exhibit the clonal allelic losses which are typical for a true neoplasm [32]. This finding is supported by the present and further studies of our group concerning the gene expression and distribution of AMPs in different lesions of the salivary glands.

Among the benign salivary gland tumours, pleomorphic adenomas are by far the most frequent adenoma of the parotid gland, whereas oxyphil adenomas and other monomorphic adenoma seem to be seldom [33]. As pleomorphic adenomas show very distinct molecular features concerning the β-defensins which differ from other benign salivary gland tumours, it seems unlikely, that cystadenolymphomas are adenomas with a lymphocytic infiltration [4-6]. A nuclear shift of the hBD-1 gene product and a decrease of hBD-1 gene expression underlines their potency for a malignant transformation into adenocarcinomas [4,5]. *In vitro* deficiency of insulin-like growth factor-1 (IGF-1) and a low basic hBD-2 and hBD-3 gene expression could be protective against a malignant transformation [6].

Because of this recent evidence that β-defensin might play an important role in the formation and malignant progression of salivary gland tumours, because of their structural and functional similarities and their gene loci on chromosome 8, the present study focused on the α-defensins. The increase of DEFA 1/3 gene expression was in pleomorphic adenomas only slight, but in cystadenolymphomas very explicit. The increase of DEFA 4 was in cystadenolymphomas 48.2-fold, but in pleomorphic adenomas there was a 3.4-fold decrease in DEFA 4 gene expression. This inversely alteration of DEFA 4 gene expression and the abundantly clear difference in DEFA 1/3 gene expression additionally contradict the hypothesis of cystadenolymphomas being adenomas with a lymphocytic infiltration. The comparable increase of DEFA 1/3 and DEFA 4 in inflamed salivary gland tissue (146.9 and 30.3-fold respectively) and cystadenolymphomas (146.9 and 48.2-fold respectively) might suggest an inflammatory aetiology and supports the theory, that cystadenolymphomas due to their polyclonal epithelial component are not a true neoplasm [32].

Pleomorphic adenomas, presenting as benign mixed tumours, are the most common neoplasms of the major salivary glands. Although benign, it is not uncommon for pleomorphic adenomas to recur, and a subset of them might undergo a malignant transformation [33]. In prior studies the authors demonstrated, that pleomorphic adenomas differ from other salivary gland tumour in their β-defensin gene expression and the cellular distribution of the hBD-1 gene product [4-6]. The down-regulation of hBD-1 gene expression is an event which could be observed in many tumour entities [16-21] and which is common in head and neck cancers also [4-6,16-21]. Additionally an up-regulation of hBD-3 was observed in oral squamous cell carcinomas (OSCC) [19,20]. This observation led to the hypothesis that in OSCCs hBD-1 works as a tumour-suppressor, whereas hBD-3 is a proto-oncogene – which could be verified by in vitro experiments [34]. In the present study pleomorphic adenomas clearly differ from other salivary gland tumours by their α-defensin gene expression as well: In comparison with healthy salivary gland tissue the increase of DEFA 1/3 gene expression was clearly in adenocarcinomas, adenoidcystic carcinomas and mucoepidermoid carcinomas, but only slight in pleomorphic adenomas. The gene expression of DEFA 4 was elevated in all lesions but only in pleomorphic adenomas there was as 3.4-fold decrease. This distribution of DEFA 4 gene expression is evocative of hBD-1 gene expression in pleomorphic adenomas and other head and neck cancers. It seems to be likely, that α-defensins as well as β-defensins are involved in tumour formation and progression of salivary gland tumours - but the interpretation of α-defensin gene expression in different head and neck lesions remains difficult and particularly inconsistent. For example in oral leukoplakia DEFA 4 is highly up-regulated (179.2-fold) whereas DEFA 1/3 does not differ much from healthy gingiva [21]. In irritation fibromas of the oral cavity it is vice versa: DEFA 1/3 is up-regulated (14-fold) and DEFA 4 is comparable to gingiva [22]. For this reason our group at the moment carefully investigates the influence of α-defensins in different head and neck tumours in vitro. To have this knowledge will help us to a better understanding of the molecular mechanisms which make a benign tumour malignant and could therefore enable us in the future to early diagnose and treat these tumours.

## Conclusions

It seems to be likely, that α-defensins as well as β-defensins are involved in tumour formation and progression of salivary gland tumours. Nevertheless the interpretation of α-defensin gene expression in different head and neck lesions remains difficult. Results from this study allow us to hypothesize that a decreased gene expression of DEFA 1/3 and 4 might protect pleomorphic adenomas from malignant transformation into adenocarcinomas. A similar expression pattern of DEFA-1/3 and -4 in cystadenolymphomas and inflamed salivary glands underlines a potential importance of immunological reactions during the formation of Warthin’s tumour.

## Competing interests

The authors declare that they have no competing interests.

## Authors' contributions

JW and MW conceived of the study and drafted the manuscript. JR carried out the immunoassays. AP participated in the sequence alignment. RR and SJ participated in the design of the figures. DK performed the statistical analysis. JPA and NN participated in the design and coordination of the study. All authors read and approved the final manuscript. HPF approved the exact diagnosis for all salivary gland tumours.

## Pre-publication history

The pre-publication history for this paper can be accessed here:

http://www.biomedcentral.com/1471-2407/12/465/prepub
